# Trivalent soluble TNF Receptor, a potent TNF-α antagonist for the treatment collagen-induced arthritis

**DOI:** 10.1038/s41598-018-25652-w

**Published:** 2018-05-09

**Authors:** Xiaofang Cui, Linmo Chang, Youwei Li, Qianrui Lv, Fei Wang, Yaxian Lin, Weiyang Li, Jonathan D. Meade, Jamie C. Walden, Peng Liang

**Affiliations:** 1 Department of Biochemistry & Molecular Biology, College of Life Sciences, Sichuan University, Chengdu, 610065 China; 2 Jining Medical University, Jining, 272000 China; 3 CloverBiopharmaceuticals, Chengdu, 610000 China; 4 GenHunter Corporation, 624 Grassmere Park, Nashville, TN 37211 USA

## Abstract

Tumor necrosis factor is a major pro-inflammatory cytokine which triggers various physiological consequences by binding to and trimerizing its receptors, and has been the single most sought-after drug target for intervening autoimmune diseases such as rheumatoid arthritis and psoriasis. However, current TNF-α blockers, including soluble receptor-Fc fusion and therapeutic antibodies, are all dimeric in structure, whereas their target TNF-α itself is homotrimeric in nature. Here we describe the development of a trivalent soluble TNF receptor and show that it is a more potent than the dimeric TNF receptor decoys in inhibiting TNF-α signaling both *in vitro* and *in vivo*. The process involves gene fusion between a soluble receptor TNFRII with a ligand binding domain and a trimerization tag from the C-propeptide of human collagen (Trimer-Tag), which is capable of self-assembly into a covalently linked trimer. We show that the homotrimeric soluble TNF receptor (TNFRII-Trimer) produced with such method is more potent in ligand binding kinetics and cell based bioassays, as well as more efficacious in attenuating collagen-induced arthritis (CIA) in a mouse model than its dimeric TNFRII-Fc counterpart. Thus, this work demonstrates the proof of concept of Trimer-Tag and provides a new platform for rational designs of next generation biologic drugs.

## Introduction

Tumor necrosis factor (TNF-α) is a major pro-inflammatory cytokine produced by human body to combat infections, and it provides necessary protections for the host^1^. However, an abnormal persistent release of TNF-α could result in autoimmune diseases, such as rheumatoid arthritis (RA), psoriasis, and others^2,3^. Hitherto, accumulative evidence has shown that TNF-α, the initial inflammatory cytokine, plays decisive roles in the occurrence and development of autoimmune diseases^4^. *In vivo*, biologically active TNF-α molecule exits as a homotrimer^1,5^, and activates the downstream signal pathway by binding to trimerized TNF-α receptors (TNFR) on the cell surface, leading to receptor trimerization and activation, which in turn triggers a cascade of events leading to inflammation^6^.

Structurally, all members of TNF family of cytokines are homotrimers^1,5^. Accordingly, fusing a soluble TNF receptor with the Fc region of immunoglobulin G1, which is capable of spontaneous dimerization via disulfide bonds, allowed the secretion of dimeric soluble TNF receptor^7^. In comparison with the monomeric soluble receptor, the dimeric TNF Receptor II-Fc fusion has a greatly increased affinity to the homo-trimeric ligand. This provides a molecular basis for the block buster anti-TNF drug Etanercept in treating rheumatoid arthritis (RA)^8^. Although Enbrel was shown to have a Ki below 1 nM to TNF-α^7^, 25 mg twice a week subcutaneous injections, which translates to μg/mL level of the soluble receptor, are required for the RA patients to achieve clinical benefits (see www.enbrel.com). The high level of recurrent Enbrel consumption per RA patients has created a great pressure as well as high cost for the drug supply, which limits the accessibility of the drug to millions of potential patients in developing countries.

**Figure 1:**
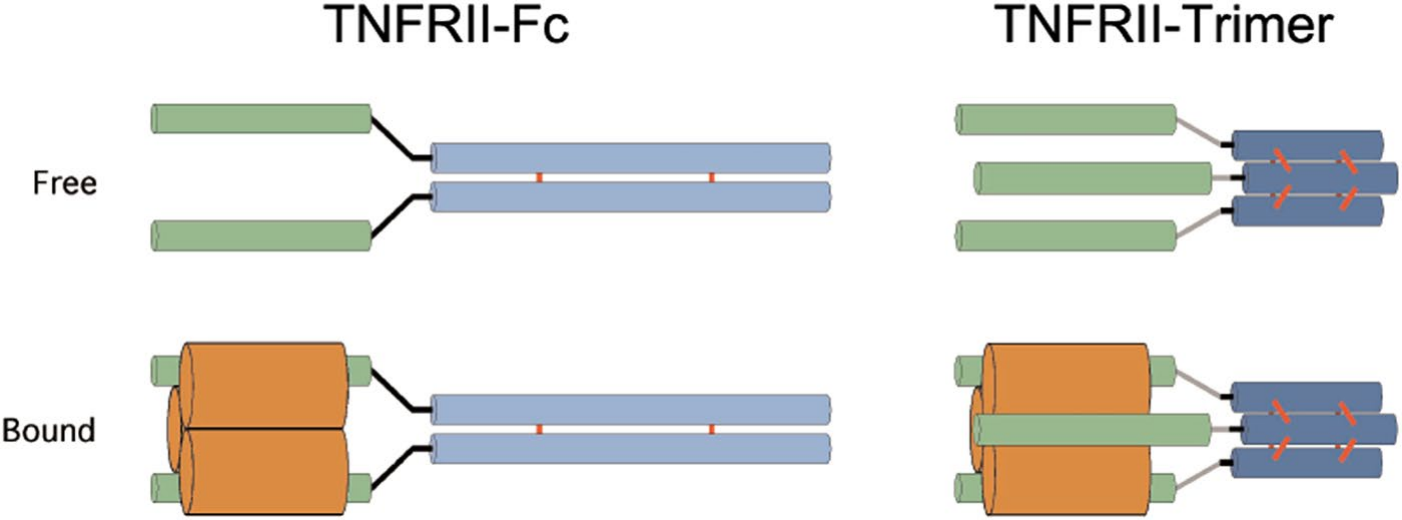
Schematic representation of the trimeric soluble human TNFRII receptor (TNFRII-Trimer) in comparison with dimeric immunoglobulin Fc fusion. On the left: Structural characteristics of a homodimeric soluble human TNFRII receptor-Fc fusion, Etanercept (TNFRII-Fc), in either ligand-free or -bound form as indicated. Domains labeled in green denote soluble TNFRII. Note that the Fc (labeled in light blue with inter-chain disulfide bonds in red) fusion protein is dimeric in structure. Given its 2-fold symmetry, the dimeric Fc fusion protein is bivalent and thus theoretically does not have the optimal conformation to bind to a homotrimeric ligand, TNF-α (labeled in brown), which has a 3-fold symmetry. On the right: Structure characteristics of the trimeric soluble human TNFRII receptor-C propeptide fusion. Given its 3-fold symmetry, the TNFRII-Trimer fusion protein is trivalent in nature, thus can perfectly dock to its trimeric ligand TNF-α. The C-propeptide of collagen capable of self trimerization is labeled in dark blue with inter-chain disulfide bonds labeled in red.

Clearly, there is a great need to be able to create secreted homotrimeric soluble receptors or biologically active proteins, which may have perfect docking sites, hence higher affinity, to their naturally occurring homotrimeric ligands, such as TNF family of cytokines. Such trimeric receptor decoys theoretically should have a much higher affinity to their trimeric ligands than their dimeric counterparts such as etanercept (Fig. 1). Such rationally designed soluble trimeric receptor analogs could increase the clinical benefits as well as potentially lower the amount or frequency of the drug injections for each patient.

Apparently, one of the feasible plans to improve the current design of the TNF-α antagonist molecule may be creating a secreted form of homo-trimeric soluble receptors, or proteins with similar biological activities, containing canonical docking sites, hence with greater affinities for the natural homotrimeric ligands than that of dimeric TNFRII-Fc. Accordingly, we have developed a soluble trimeric TNFR antagonist which acquires greater affinity to its natural homo-trimeric ligands in the hope for a significant increase of its clinical benefits.

To be therapeutically feasible, a desired trimerizing protein moiety for biologic drug designs should fulfill the following criteria. Ideally it should be part of a naturally secreted protein, like immunoglobulin Fc, and abundant (non-toxic) in circulation, human origin (lack of immunogenicity), relatively stable, capable of efficient self-trimerisation and pertain an optimal geometry in projecting soluble receptor to be trimerized to ensure maximum ligand binding. Collagen, being the most abundant protein in human body, is a major component of the extracellular matrix^9^. The fibrillar types of collagen are synthesized in soluble forms of trimeric precursors procollagen, whose central triple-helical domains consist of hundreds of uninterrupted “G-X-Y” repeats (orglycine repeats), and the N- and C-terminal are flanked by non-collagenous propeptide domains^10^. The assembly of a trimeric pro-collagen molecule is initiated by the association of the C-propeptide domains via a single type of inter-chain disulfide bond formed between Cys47 and Cys64 among neighboring chains^11^, and the triple helix folds and moves toward the N-terminus in a zig-zag-pattern^10,12^. Following by the completion of its assembly, the collagen maturation requires an additional step, the proteolytic removal of the C- and N-terminal extensions. It is revealed that the C-propeptide domains are cleaved off by BMP-1 proteinases^13,14^, which recognizes a specific peptide sequence embedded in the border region between the glycine repeats and C-prodomain.

Recently we have demonstrated the use of the C-prodomain of human α1(I) collagen to form covalently linked TRAIL which is a member of TNF family of cytokines and showed it was fully activate in biological functions as the native non-covalently linked trimeric ligand, whereas the dimierc TRAIL-Fc fusion retained little activities^15^.

Here we use the C-propeptide domain of human type III procollagen as a trimierzation tag for TNFRII. We provide evidence that the homotrimeric soluble TNF receptor (TNFRII-Trimer) produced with such method is more potent both in ligand binding kinetics and cell based bioassays *in vitro*, as well as more efficacious in attenuating collagen-induced arthritis (CIA) in an mouse model *in vivo* than the dimeric TNFRII-Fc fusion protein.

## Results

### High-level expression and purification of TNFRII-Trimer fusion protein

**Figure 2:**
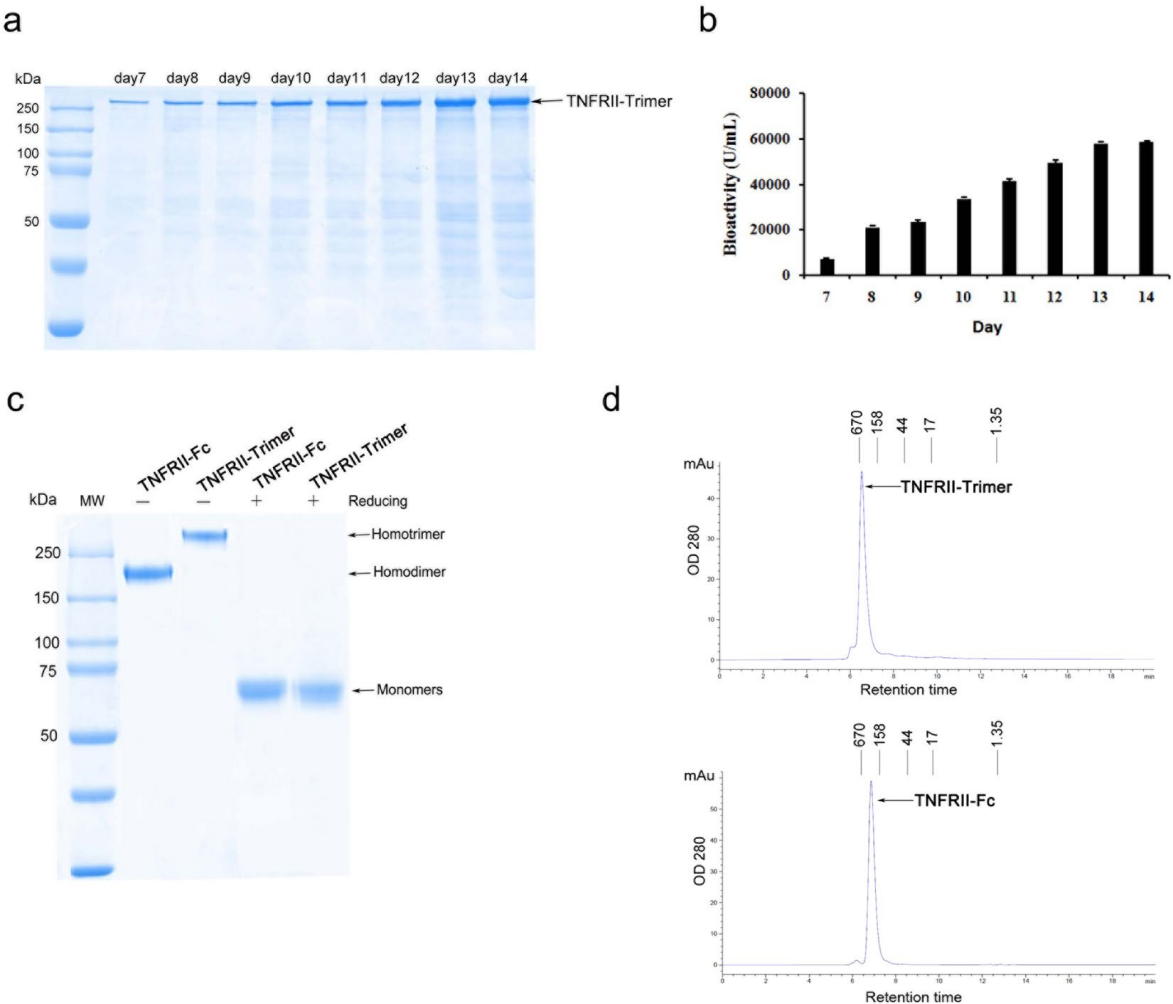
High-level expression and purification of TNFRII-Trimer fusion protein. (**a**) 10% SDS-PAGE analysis of TNFRII-Trimer expression from a fed-batch serum-free cell culture in the bioreactor. 10 μl of cell-free conditioned medium from day 7 to day 14 were analyzed under non-reducing condition followed by Coomassie brilliant blue staining. (**b**) Bioassay analysis of TNFRII-Trimer production in conditioned medium from day 7 to day 14. (**c**) SDS-PAGE analysis of purified TNFRII-Trimer under either non-reducing or reducing conditions with TNFRII-Fc as a control. TNFRII-Fc (2 μg) and purified TNFRII-Trimer (2 μg) were analyzed by a 10% SDS-PAGE and stained with Coomassie Blue. (**d**) Purity evaluation of TNFRII-Trimer by SEC-HPLC, with OD280 detection. TNFRII-Fc served as a control. The main peak area of TNFRII-Trimer was 95%.

To obtain sufficient amounts of recombinant TNFRII-Trimer fusion protein with high purity for functional analysis, we screened for high-titer production clones of TNFRII-Trimer vector-transfected CHO cells via MTX-mediated gene amplification. The resulting leading clone was adapted to serum-free medium and fed-batch cell culture from bioreactor led to high-level expression of TNFRII-Trimer (Fig. 2a). During the course of the cell culture process, conditioned media from each day were taken to assess the bioactivity of TNFRII-Trimer using a TNF-α-sensitive cell line-L929 by MTT staining (Fig. 2b). Results clearly indicated that bioactivity increased over time as the production of TNFRII-Trimer continued. To obtain the TNFRII-Trimer in purified form, TNFRII-Trimer from serum-free conditioned medium was purified to near homogeneity via a Blue Sepharose chromatography under step-wise salt gradient elution. The purified TNFRII-Trimer was characterized by SDS-PAGE under either non-reducing or reducing conditions followed by Coomassie blue staining (Fig. 2c). TNFRII-Fc served as a control for dimeric TNFRII linked by disulfide bonds formed between the Fc domains. The result clearly revealed that TNFRII-Trimer formed a disulfide bond-linked homotrimer as predicted by its higher molecular weight under non-reducing condition and by size-exclusion HPLC (SEC-HPLC) than that of TNFRII-Fc (Fig. 2c,d) and its purity reached over 95% (Fig. 2d).

### Physicochemical characterizations of TNFRII-Trimer

**Figure 3:**
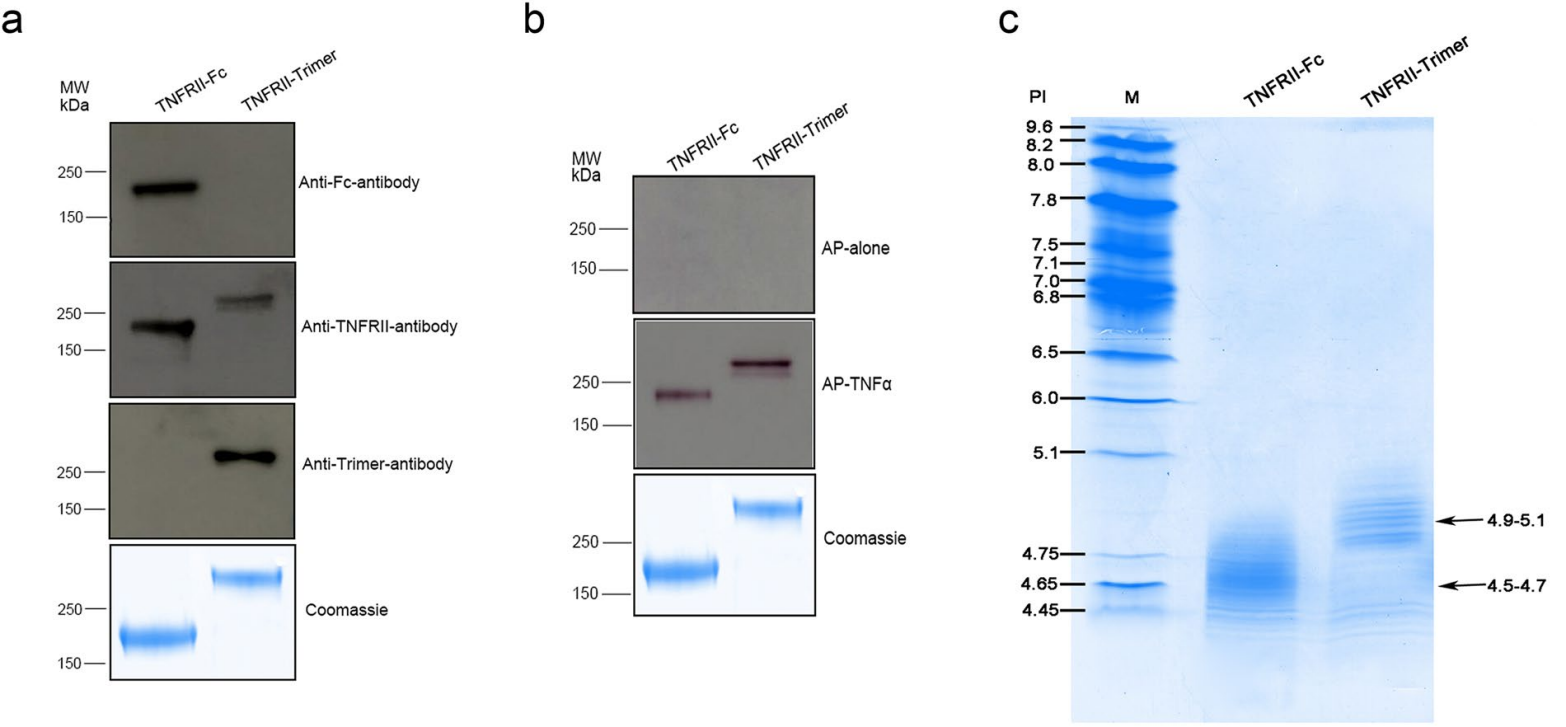
Biochemical characterization of purified TNFRII-Trimer. (**a**) TNFRII-Trimer was analyzed by Western blot under non-reducing conditions to confirm the size and integrity of the fusion protein. 0.2 μg of TNFRII-Trimer and TNFRII-Fc were analyzed by western blot using antibody against TNFRII-domain, Trimer-domain and Fc-domain, respectively. Full-length blots are included in the Supplementary information (**a**). (**b**) Ligand-receptor affinity staining analysis of TNFRII-Trimer binding to AP-tagged ligands (AP-TNF-α). TNFRII-Fc (2 μg) and TNFRII-Trimer (2 μg) were each separated by non-reducing SDS-PAGE and either visualized by Coomassie brilliant blue staining or transferred to a PVDF membrane, followed by *in situ* detection with AP-TNF-α. Both TNFRII-Trimer and TNFRII-Fc were detected by their AP-tagged ligands (AP-TNF-α) but not by AP alone (negative control). Full-length blots are included in the Supplementary information (**b**). (**c**) IEF was performed in fusion protein TNFRII-Trimer and TNFRII-Fc. The PI value of TNFRII-Trimer and TNFRII-Fc is shown, respectively. M represented marker.

To verify the structural feature and integrity of TNFRII-Trimer fusion protein vs TNFRII-Fc, antibodies specific to human TNFRII-domain, Fc-domain and the Trimer-Tag -domain were used to conduct the western blot analysis, respectively. As expected, the presence of TNFRII and Trimer-Tag domains of the trimeric TNFRII-Trimer fusion protein were each confirmed by the respective antibodies. As a negative control, the Trimer-Tag-specific antibody failed to detect TNFRII-Fc, while the Fc-specific antibody failed to detect TNFRII-Trimer fusion protein under non-reducing conditions (Fig. 3a). The result indicated that the TNFRII-Trimer existed essentially as a covalently-linked homotrimer as predicted.

Previously we have discovered that members of TNFR superfamily of receptors are thermo-stable for their ligand binding^16^ under *in situ* ligand-receptor binding assays after SDS-PAGE separation and transfer of the purified TNFRII fusion proteins to a PVDF membrane. The result indicated that under non-reducing condition, TNFRII-Trimer, like the TNFRII-Fc fusion protein could bind to its AP-tagged ligand, TNF-α (AP-TNF-α), whereas AP alone failed to do so (Fig. 3b). These data suggested that TNFRII-Trimer retained its biological functions for ligand binding.

Isoelectric focusing (IEF) was performed to identify charge variants of TNFRII-Trimer fusion proteins. The result showed that the isoelectric point of TNFRII-Trimer was within 4.3–5.0 and that of TNFRII-Fc was within 4.5–4.8 (Fig. 3c). Compared with that of TNFRII-Fc, the pI of TNFRII-Trimer fusion protein was slightly basic.

To determine whether TNFRII-Trimer was properly glycosylated, the resorcinol method was applied to detect sialic acid (SA) content of TNFRII-Trimer with TNFRII-Fc as a control. The SA content values for TNFRII-Trimer and TNFRII-Fc were determined to be 35.13 and 31.05 mol of sialic acid/mol of protein, respectively (Table 1), indicating heavy glycosylation in both fusion proteins.

**Table 1:** Sialic acid (SA) estimation was performed by resorcinol methods.

Sample	OD at 580 nm	mol of sialic acid/mole of protein (mol/mol)
TNFRII-Fc	0.024	31.05 ± 0.73
TNFRII-Trimer	0.009	35.13 ± 0.57

Note: each sample was measured in triplicate, and took the mean to eradicate any discrepancies. Data was shown in Mean ± S.E.M.

### Neutralization of TNF-α-induced cytotoxicity by TNFRII-Trimer

**Figure 4:**
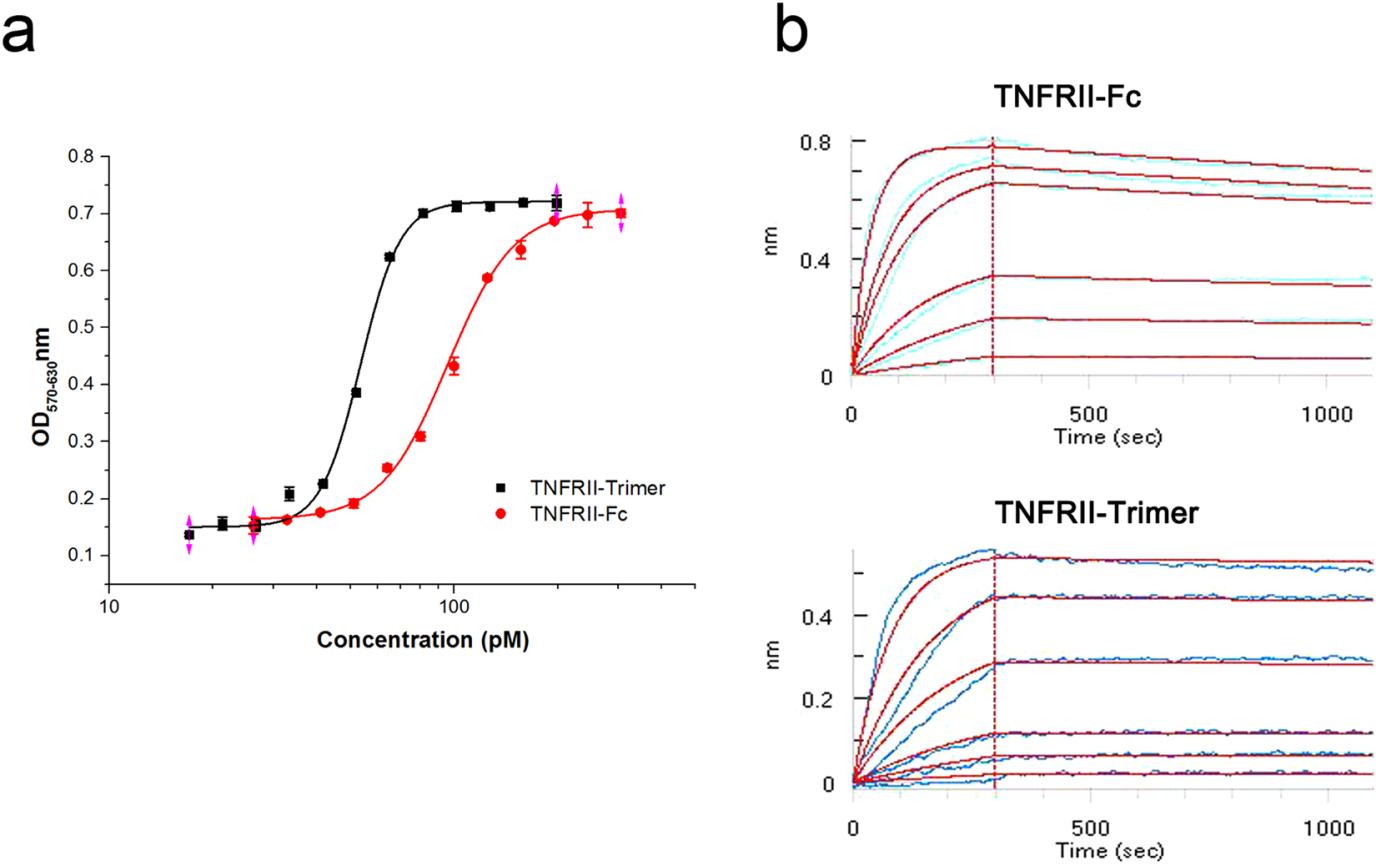
Bioactivity and affinity of TNFRII-Trimer and TNFRII-Fc to TNF-α. (**a**) Bioactivity detection of TNFRII-Trimer and TNFRII-Fc. Neutralizing TNF-α induced-cytotoxicity assays were performed using L929 cell line, and cell viability was evaluated using a colorimetric MTT assay. The IC_50_ value of TNFRII-Trimer and TNFRII-Fc is 54pM and 98.6pM, respectively. (**b**) Ligand binding kinetics of TNFRII-Trimer and TNFRII-Fc binding to TNF-α were assessed by biolayer interferometry measurements. The Super Streptavidin biosensor tips of the ForteBio Octet RED 96 were coated with biotinylated TNFRII receptors. The biosensor tips were dipped in increasing concentrations gradient of the same molarity (2.5 nM–39 nM) to measure binding of TNFRII-Trimer and TNFRII-Fc to TNF-α and subsequently moved to wells containing buffer (PBS) to measure dissociation rates. Detailed results were shown in Table 2.

To determine if TNFRII-Trimer is a potent inhibitor of the trimeric TNF-α, an *in vitro* TNF-α bioassay was carried out using a TNF-α sensitive cell line L929^17^. The result shown in Fig. 4a indicated that TNFRII-Trimer had higher activities than that of TNFRII-Fc in inhibiting the TNF-α mediated apoptosis. The IC_50_ values for TNFRII-Trimer and TNFRII-Fc based weight basis were 8.5 and 10.1 ng/mL, respectively, according to the dose-response cell index curve (Fig. 4a). Based on theoretical molecular weight of the two native TNFRII fusion proteins, the TNFRII-Trimer with each subunit having a molecular weight of 52.5 kDa is at lease 1/3 larger than the dimeric TNFRII-Fc with each subunit being 51.2 kDa in molecular weight (Fig. 2c,d). Therefore, based on molar ratio, the neutralization activities of trimeric TNFRII-Trimer was about 2-fold higher than that of the dimeric TNFRII-Fc with IC_50_ being 54.0pM and 98.6pM, respectively. Furthermore, the much steeper S-curve in concentration dependent TNF-α neutralization for TNFRII-Trimer observed in comparison with TNFRII-Fc (Fig. 4a) was also indicative of tighter and cooperative binding of each subunit of TNFRII-Trimer to the ligand.

### Kinetic analysis of TNFRII-Trimer binding to human TNF-α

To determine the molecular binding kinetics of TNFRII-Trimer to TNF-α vs TNFRII-Fc, Fortebio biolayer interferometry measurement was employed. Biotin-labeled TNFRII-fusion proteins were first captured on Streptavidin (SA) sensors, and real-time binding curves were obtained by exposing the biosensor to gradient concentrations of TNF-α. As expected, TNFRII-Trimer exhibited higher ligand binding affinities than that of TNFRII-Fc with KD being 7.39 × 10^−11^ M vs. 23.1 × 10^−11^ M, respectively. The binding kinetics parameters were listed in Table 2. While TNFRII-Fc bound to TNF-α with K_on_ = 6.16 × 10^5^ M^−1^ s^−1^, which was approximately twice faster than TNFRII-Trimer (K_on_ = 3.28 × 10^5^ M^−1^ s^−1^), the latter dissociated (K_off_ = 2.42 × 10^−5^ s^−1^) from TNF-α upon binding at a rate about six times slower than that of the dimeric TNFRII-Fc (K_off_ = 14.2 × 10^−5^ s^−1^).

**Table 2:** Assessment of kinetic rate constants for binding to TNF-α by Fortebio.

Sample	K_on_ (M^−1^ s^−1^)	K_dis_ (s^−1^)	K_D_ (M)
TNFRII-Fc	6.16 × 10^5^	14.2 × 10^−5^	2.31 × 10^−10^
TNFRII-Trimer	3.28 × 10^5^	2.42 × 10^−5^	7.39 × 10^−11^

### TNFRII-Trimer ameliorated the severity of arthritis in a DBA/1mouse CIA model

Having demonstrated that TNFRII-Trimer had higher affinity in ligand binding as well as exhibiting higher inhibitory effects than dimeric TNFRII-Fc *in vitro* by a TNF-α bioassay, we then set out to test whether TNFRII-Trimer could be therapeutically efficacious in the collagen-induced arthritis.

**Figure 5:**
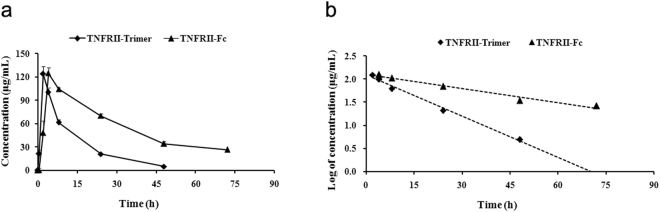
Pharmacokinetics analysis of TNFRII-Trimer and TNFRII-Fc in DBA/1 mice. (**a**) Plasma concentrations versus time profile of TNFRII-Trimer after i.p. administration of TNFRII-Trimer and TNFRII-Fc in DBA/1 mouse. (**b**) Plasma concentration (log-scale) versus time profile of TNFRII-Trimer and TNFRII-Fc after i.p. administration in DBA/1mouse. Data represented mean ± SD (n = 3).

To determine the dosing strategy of pharmacodynamic study, we first conducted a pharmacokinetic study of TNFRII-Trimer in DAB/1 mice, and TNFRII-Fc was also tested in parallel for comparison. The results indicated that TNFRII-Trimer and TNFRII-Fc when administrated via *i*.*p*., with dosages being both at 12.5 mg/kg showed similar pharmacokinetic profiles as shown in the drug concentration-time profile in semi-logarithmic scale (Fig. 5a,b). A mono-compartment model best describe the pharmacokinetic profiles of them in the first-order elimination processes. As shown in Fig. 5a, a rapid absorption with the T_max_ of 2 h and mean maximum concentrations (C_max_) of 123 ± 10.09 μg/mL was observed, following a single i.p injection. In contrast, TNFRII-Fc had a T_max_ of 4 h and C_max_ of 125 ± 7.14 μg/mL. The half-lives of TNFRII-Trimer and TNFRII-Fc in DBA/1 mice were determined to be 10 hours and 30 hours, respectively by subjecting the individual slope of line into the formula of first order kinetics.

**Figure 6:**
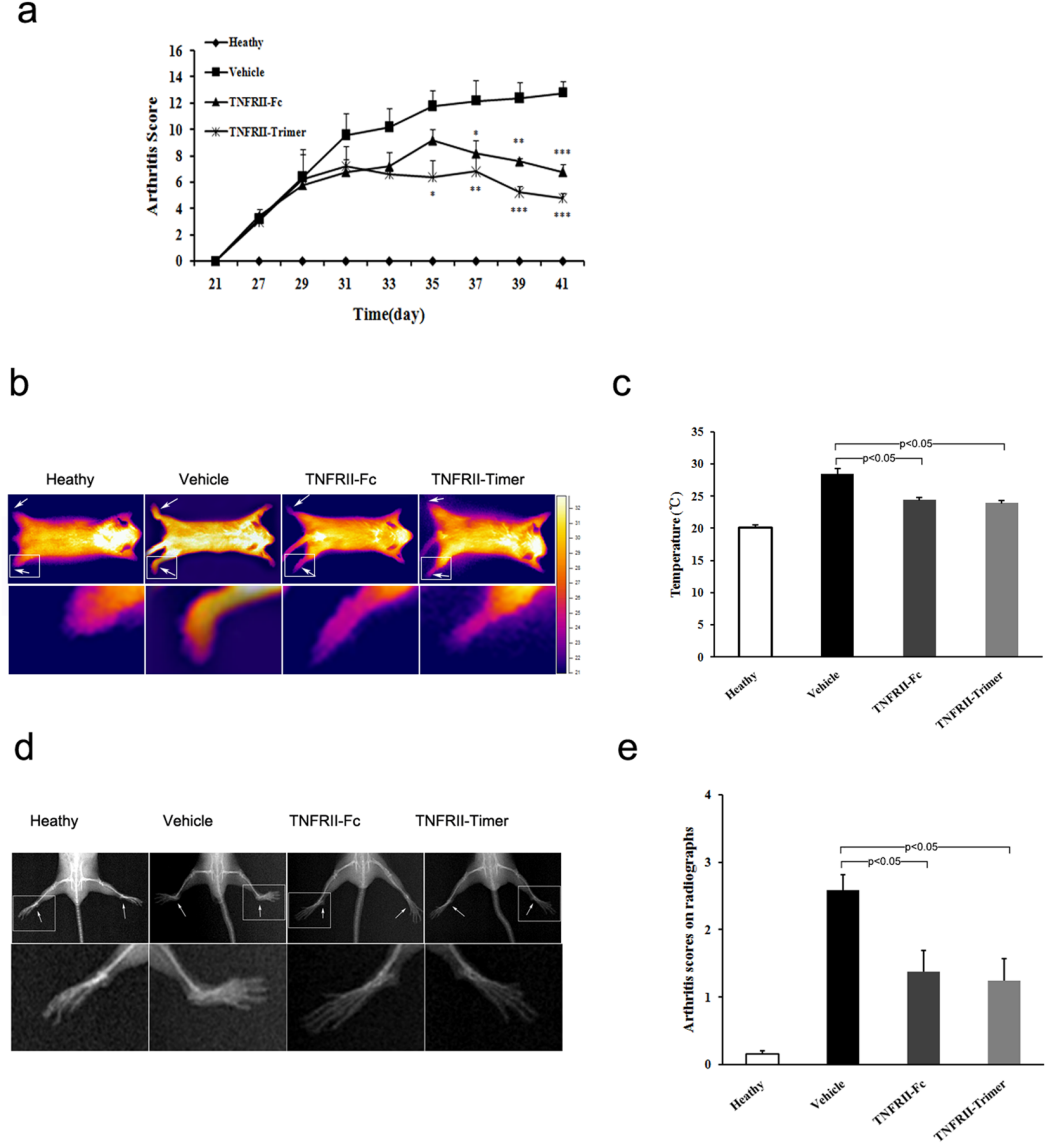
Inhibition of established CIA by TNFRII-Trimer. (**a**) Comparison of arthritis scores from DBA/1 mice treated with either TNFRII-Trimer or TNFRII-Fc. The results were representative of four independent observers. *p < 0.05, **p < 0.01, ***p < 0.001 (**b**) Representative images of infrared thermography (IRT) of the hind paws of the CIA mice on day 41. In comparison with healthy control and treatment groups, hind paws from vehicle group of mice showed significantly higher temperature and severe swelling (indicated by arrows). (**c**) Comparison of hindpaws’ temperature from DBA/1 mice treated with either TNFRII-Trimer or TNFRII-Fc. Regional temperature was analyzed by XJ-Infrared report analysis software. Values were mean ± SD (n = 5). (**d**) Representative images of radiograph of the hind paws of CIA mice on day 41. As compared with healthy control and treatment groups, hind paws from vehicle group of mice were significantly deformed, swollen, and with joint space narrowing (indicated by arrows). (**e**) Comparison of the degree of joint swelling and bone erosion from DBA/1 mice treated with either TNFRII-Trimer or TNFRII-Fc. Values were mean ± SD (n = 5). The results were from a representative experiment.

In order to accurately compare the therapeutic efficiencies of the dimeric and trimeric TNFRII fusion protein, same dosing strategy of two drugs with equal dosage (12.5 mg/kg) and dosing frequency (once every two days) was employed in pharmacodynamic study. The result showed that treatment of CIA mice with TNFRII-Trimer led to significant reduction in the severity scores of arthritis by more than half compared to non-treatment control group (p < 0.05) (Fig. 6a). In a side-by-side comparison with the positive control TNFRII-Fc, TNFRII-Trimer exhibited also statistically more potent effect by nearly 2 basic points in arthritis index in the treatment of established CIA than TNFRII-Fc (Fig. 6a). The measurements in clinical arthritis severity scores were confirmed with both infrared and radiography analysis, which supported the pharmacodynamic functions of TNFRII-Trimer in suppressing the severity of joints damage for CIA mice (Fig. 6b–e).

To further evaluate the therapeutic effect of dimeric and trimeric TNFRII fusion proteins in the mouse CIA model, paraffin-embedded tissues from DBA/1 mice were sectioned and analyzed by both H/E and *in situ* ligand-receptor functional staining using AP-tagged TNF-α (AP-TNF-α) (in Supplementary Fig. 1). H&E staining clearly showed at histological level that the paws from mock treated mice appeared severely inflamed in a side-by-side comparison with that of healthy mice. In contrast, both TNFRII-Trimer and TNFRII-Fc treatment led to marked attenuation of the degree of swollenness of the digits from the paws of the animals. Moreover, compared to normal control mice, disease tissues from the swollen paws of CIA mice exhibited a large number of immune infiltrates that were stained strikingly positive for AP-TNF-α. In contrast, paws from both dimeric and trimeric TNFRII treatment groups exhibited significantly reduced number of immune infiltrates (Supplementary Fig. 1). We have recently shown that AP-TNF-α specifically binds to TNF receptor positive cells^16^ and that these immune infiltrates from CIA mice were in fact macrophages via co-staining for F4/80^18^.

The potential toxicity of TNFRII-Trimer was evaluated using histological analysis of major organ tissues from mice (Supplementary Fig. 2). Compared with the normal control, organ tissues from TNFRII-Trimer treated animals did not show any noticeable morphological aberrations. Further analysis of hepatotoxicity of TNFRII-Trimer on human hepatocytes was also conducted using cultured LO_2_ cells, which showed no adverse effects on cell viability and apoptosis (Supplementary Fig. 3). These results suggest that TNFRII-Trimer has a good safety profile in the animals and as well as to human hepatocytes.

## Discussion

Tumor necrosis factor (TNF-α is one of the most potent mediators of the inflammatory response and its aberrant expression plays prominent roles in the pathogenesis of autoimmune disorders^1,19,20^. In fact, TNF/TNFR system represents the single largest and validated therapeutic target in the clinics, accounting for major chronic autoimmune diseases, such as rheumatoid arthritis and psoriasis^21^.

Since TNF-α is homotrimeric in nature, current anti-TNF biologics with a two-fold symmetry, such as TNFRII-Fc, may not perfectly dock to its homotrimieric ligand with a three-fold symmetry (Fig. 1). To develop a more potent TNF antagonist with improved efficacy, a trimeric TNFRII fusion protein, TNFRII-Trimer, was constructed and characterized in this study. Previously a novel trimeric soluble fusion protein, Trail-Trimer with C-propeptide of type I collagen as the trimeric foldon was successfully expressed in a mammalian (CHO) expression system in our laboratory^15^. Since type I collagen is mainly a heterotrimer triple-helical molecule consisting of two α-1 chains and one α-2 chain, whereas homotrimeric isoform of type I collagen consisting of three α-1 chains, is only found in fetal tissues, fibrosis, and cancer in humans^22^. Here employed the trimeric domain from the C-propeptide of type III collagen which forms exclusively homotrimer. To test whether the TNFRII-Trimer can be successfully secreted as a homotrimeric soluble receptor or biologically active fusion protein, we first developed a CHO cell line and complementary serum free cell culture process from which TNFRII-Trimer can be produced at high level. The resulting TNFRII-Trimer was highly purified from the cell culture media and used for further characterization. In order to verify the structural validity and bioactivity of the trimeric TNFRII-Trimer *in vitro*, we conducted a series of physical-chemical characterizations, and compared them with that of TNFRII-Fc in a side-by-side analysis. Structural studies of TNFRII-Trimer revealed that the soluble TNFRII-Trimer fusion protein was indeed a disulfide bond-linked homotrimer, consists of both TNFRII and type III collagen C-prodomain with heavy glycosylation. More importantly, using AP-tagged ligand, direct binding of TNF-α to TNFRII-Trimer was evident even with denatured trimeric soluble receptor protein separated on an SDS-PAGE as predicted by our previously discovery^16^.

L929 cell-based bioassay for TNF-α confirmed that TNFRII-Trimer could effectively bind to the TNF-α, thereby blocking the ligand induced cell apoptosis. In a side-by-side comparison, the trimeric soluble TNFRII-Trimer had an apparent IC_50_ twice lower than that of dimeric TNFRII-Fc, based on molar ratio of each native fusion protein. Furthermore, the apparent and much steeper S-curve in a concentration-dependent TNF-α neutralization for TNFRII-Trimer, in comparison with TNFRII-Fc, was also indicative of tighter and cooperative binding of each subunit of TNFRII-Trimer to its ligand.

Molecular binding kinetics studies with Fortebio biolayer interferometry measurement confirmed the finding of cell-based bioassay. The KD of the trimeric TNFRII-Trimer (7.39 × 10^−11^ M) for human TNF-α ligand was over three times lower than that of the dimeric TNFRII-Fc (23.1 × 10^−11^ M). The increased affinity was mostly attributed to nearly 6 times slower in off rate for the bound ligand. These results confirmed our hypothesis that trimeric TNF receptor fusion protein had a better symmetry, thus tighter binding to its homotrimeric ligand, TNF-α.

The therapeutic efficacy for TNFRII-Trimer *in vivo* was also evaluated using the standard mouse CIA model. Based on the pharmacokinetic studies, the TNFRII-Trimer had a half-life (10 hr) 3 times shorter than that of TNFRII-Fc in DBA/1 mice. While this attribute is disadvantageous, if one considers the *in vitro* potency for TNFRII-Trimer (in the range of ng/mL) in blocking TNF-α, and that each mouse contains approximately 2 mL of blood, dosing at 12.5 mg/kg per animal would result in initial drug concentration of around 125 μg/mL, which would attenuate to about 4.5 μg/mL 48 hr TNFRII-Trimer. This concentration is nearly three orders of magnitude higher than its IC_50_ *in vitro*. Therefore, identical dosing strategy was employed to compare TNFRII-Trimer and TNFRII-Fc pharmocodynamics studies. Consistent with our prediction, the trimeric soluble TNFRII exhibited better efficacy in suppressing disease severity compared with TNFRII-Fc. The efficacy of TNFRII-Trimer in arthritis treatment were further verified at the levels of tissue histology. Both morphological and pathological examination of immune infiltrates in the paraffin-embedded disease tissues revealed that the severity of arthritis was significantly ameliorated in mice treated with TNFRII-Trimer. Besides, our study also demonstrated that TNFRII-Trimer was safe to animals and without any hepatocyte toxicity.

Taken together, we have demonstrated that with the TRIMER tag platform from pro-collagen, any soluble receptors or secreted proteins can be efficiently trimerized into disulfide bond-lined trimmers with either type I and type III collagen C-prodomain, and expressed as secreted proteins. Such trimeric fusion proteins are trivalent in structure with a 3-fold symmetry and thus may have superior biological properties than that of either naturally occurring or existing biologic proteins. Such soluble trimeric human TNF receptors may prove to be more effective than the current dimeric soluble TNF receptor (e.g. Enbrel) or therapeutic antibodies on the market in treating autoimmune diseases such as RA. Future clinical trials should provide such pivotal information.

Although it is generally believed that TNF family of ligands are mostly homotrimeric in solution or based on x-ray crystallography, biological functional assays and structural analysis of recombinant TNF expressed in bacteria suggested that both trimeric and dimmeric forms of the ligand could co-exist^23^. Thus, in future experimentation, it will be also interesting to see if the trimeric and dimeric TNF blockers can have a synergistic effect in blocking TNF signaling in both animal models and in the clinic for treating autoimmune diseases.

Although TNF antagonists have been the mainstay for the treatment rheumatoid arthritis, new venues of therapeutic strategies, such as the induction of immune tolerance^24,25^, blockade of other inflammatory cytokines such as GM-CSF signaling pathways^26,27^ and immune co-stimulators such as OX40L^28^, are being explored. Combinatory therapies from the above that eventually restore the normal cytokine crosstalk that went haywire in autoimmune diseases such as RA may lead to synergistic therapeutic effect or eventual cure of the disease.

## Methods

### Construction of pTRIMER expression vector, expression and purification of TNFRII-Trimer fusion protein

The cDNA sequence encoding the entire C-propeptides of human α1 (III) collagen containing no glycine-repeat with a mutated BMP-1 recognition site was amplified by PCR using EST clones purchased from the American Type Culture Collection (ATCC, Manassas, VA, USA). The amplified cDNA was cloned into the pAPtag-2 mammalian expression vector (GenHunter Corp, Nashville, TN, USA), replacing the AP coding region, and creating the resulting pTRIMER (T3 version) vector. The cDNA sequence coding the entire soluble human TNFRII (aa1–256) without the trans-membrane and cytoplasmic domain was amplified by PCR and cloned into either Hind III or BglII sites of pTRIMER expression vectors to allow in-frame fusion with the TRIMER tag (C-propeptide), so TNFRII-Trimer is expressed as a secreted protein. pTNFRII-Trimer expression vector was stably transfected into GH Chinese hamster ovary CHO (dhfr−/−) cell line (GenHunter, Nashville, TN) using FUGENE 6 (Roche, Mannheim, Germany) grown in IMDM with 10% FBS and 1% penicillin-streptomycin supplemented with HT (Sigma, St, Louis, MO, USA). After stepwise gene amplification with increasing concentrations (0–0.5 μM) of MTX (Sigma, Nashville), the clone with highest TNFRII-Trimer titer determined by bioassay with a TNF-α sensitive cell line L929 was obtained. The cells were then adapted to SFM-4-CHO (Hyclone, Logan, UT, USA) serum-free medium, and TNFRII-Trimer was produced in a lL Celligen bioreactor (Eppendorf, Ontario, Canada) under Fed-batch process with Cell Boost 2 (Hyclone, Ontario, Canada) added every other day from day 3 until harvest on day14. The TNFRII-Trimer titer was monitored daily with SDS-PAGE. After 14 days of culturing in bioreactor, TNFRII-Trimer was purified to homogeneity from the conditioned medium using HiTrap BlueHP (Blue Sepharose) (GE Healthcare, Logan, UT, USA) under a salt gradient elution, according to the manufacturer’s instructions. The purity of TNFRII-Trimer was determined by both SDS-PAGE and SEC-HPLC^29^ (Sepax Zenix-C SEC. 300, Newark, DE, USA). Purified TNFRII-Trimer was ultra-filtrated into PBS before being used for further characterization.

### Western blot and ligand affinity blot analysis

Antibodies recognize the Trimer-Tag domain and the TNFRII-domain of TNFRII-Trimer and human Fc-domain of TNFRII-Fc were used for their characterizations in this study. They were Rabbit anti-Trimer (CloverBiopharmaceuticals, Chengdu, China), mouse anti-TNFRII (mab226, R&D System) and horseradish peroxidase (HRP) coupled goat anti-human Fc (Southern Biotech, Birmingham, AL, USA), respectively. Purified TNFRII-Trimer (0.2 μg) and TNFRII-Fc (CloverBiopharmaceuticals, Chengdu, China) (0.2 μg) were analyzed by western blot on a 10% SDS-PAGE under non-reducing (-mercaptoethanol) condition using the above three antibodies to verify the Trimer-tagged TNFRII. The ligand-receptor affinity blot analysis was carried out using AP assay reagent S (GenHunter) following the manufacturer’s instructions as previously described^30^. Two μg of purified TNFRII-Trimer and TNFRII-Fc were separated on a 10% SDS-PAGE under non-reducing condition and either visualized by Coomassie Blue staining or transferred to PVDF membranes (GenHunter). The blots were then probed with 1 U/mL of either AP alone or AP-TNF-α (CloverBiopharmaceuticals, Chengdu, China) fusion protein, followed by visualization with AP Assay Reagent S (GenHunter).

### Isoelectric focusing analysis and sialic acid estimation

Isoelectric focusing was performed using BioRad horizontal IEF apparatus following the manufacturer’s manual instructions. The pH gradient of precast IEF gel from Lonza was 3.0–10.0 and TNFRII-Trimer was visualized by Coomassie Brilliant Blue R-250. The sialic acid content of TNFRII-Trimer and commercial TNFRII-Fc were detected by resorcinol method. Purified TNFRII-Trimer and commercial TNFRII-Fc were mixed with resorcinol-hydrochloric acid solutions by boiling water bath for 30 minutes. After cooled, butanol-butyl acetate mixture (12:48) were used to extracting the test substance and absorbance of the upper phase was measured at a test wavelength of 580 nm by a wavelength scanner (Beckman). The sialic acid content of the fusion proteins was calculated using the formula of standard curve generated from the standards of sialic acid.

### Bioassay, binding kinetics and affinity measurements

The biological activity of TNFRII-Trimer and commercial TNFRII-Fc were assessed by a MTT tetrazolium (Sigma) cytotoxicity assay as described previously^17^. Briefly, L929 cells (ATCC)were seeded in 96-well plates at a density of 2 × 10^4^ cells/well. After overnight culture, L929 cells were treated with 1.5-fold serial dilutions of either TNFRII-Trimer or commercial TNFRII-Fc with 2 μg/mL actinomycin D (Sigma) and 2.5 ng/mL human TNF-α (R&D Systems). After 16 hours of incubation at 37 °C, 20 μL of 5 mg/mL MTT was added and further incubated for 4 hours. After aspirating the supernatant media from the wells, 100 μL of DMSO (Sigma) was added to dissolve Formazan crystal. The absorbance at a test wavelength of 570 nm and a reference wavelength of 630 nm was recorded.

Binding kinetics measurements were performed using Bio-Layer Interferometry on FortéBio Octet QKe instrument (Pall, New York, NY, USA)^31^. Prior to kinetics measurements, both TNFRII-Trimer and commercial TNFRII-Fc were biotinylated with NHS-PEG4-biotin kit (Thermo Fisher Scientific) following the manufacturer’s instructions. Unbound NHS-PEG4-biotin was removed via ultra-filtration in PBS. For kinetic analysis of biotinylated TNFRII-Trimer and commercial TNFRII-Fc with TNF-α, soluble TNFRII-Trimer and commercial TNFRII-Fc (10 μg/mL) were immobilized on streptavidin (SA) biosensors (Pall) for 300 s to ensure saturation. The 96-wellmicroplates used in the Octet were filled with 200 μL of sample or buffer per well. Following a washing step, association and dissociation measurements were carried out using serial dilutions of TNF-α (R&D Systems). The accurate kinetic parameters (K_off_ and K_on_) and affinity constant (KD) were calculated using the Octetsoftware9.0 provided by the manufacturer.

### Pharmacokinetics and pharmacodynamics studies of TNFRII-Trimer in CIA mouse model

Six-week old male DBA/1 mice were purchased from Beijing HFK Bioscience and kept under standard pathogen-free conditions in the animal care center at Sichuan University and received humane care. All animal experiments were approved by The Institutional Animal Care and Use Committee (IACUC) in Sichuan University and were conducted according to international guidelines for animal experimentation.

For pharmacokinetics study, six animals were randomly divided into two groups, three in each group. Each of the three mice was dosed at 12.5 mg/kg with either TNFRII-Trimer or commercial TNFRII-Fc via i.p injections. Sera samples were collected from mouse tails at different time points after drug administration.

Serum drug levels of TNFRII-Trimer and commercial TNFRII-Fc were determined by ELISA assays. Briefly, diluted plasma samples from mice treated with TNFRII-Trimer or commercial TNFRII-Fc were added into each well of 96-well ELISA plates (Maxsorp, Corning) which were previously coated with 1 μg/mL of human TNF-α (R&D). For TNFRII-Trimer group, following 1 hour of incubation with Rabbit anti-Trimer antibody (Clover Biopharmaceuticals, Chengdu, China), signals were detected with Goat anti-rabbit-IGg conjugated HRP (Southern Biotech, Birmingham, AL, USA). For TNFRII-Fc group, Goat anti-human IgG conjugated HRP (Southern Biotech, Birmingham, AL, USA) was used to detect the signals. The absorption value in 450 nm wavelength was measured with TMB-ELISA Substrate Solution (Thermo Scientific). Serum drug concentrations were calculated by using the formula of standard curve, which obtained from purified TNFRII standards. Ke was evaluated by least square regression of the points describing the terminal log-linear decaying phase (T_1/2_ = ln2/Ke). The areas under TNFRII-Trimer and commercial TNFRII-Fc concentrations versus time curves (AUC) were calculated by Graph Pad prism6.0 software.

For pharmacodynamics study, the establishment of mouse model for CIA followed the protocol with two immunizations and treatment regimens^32^. Mice were randomly divided into 3 treated groups and one healthy control group (n = 5). Mice in each treatment group were received 200 μL of either PBS or TNFRII-Trimer (12.5 mg/kg) or commercial TNFRII-Fc (12.5 mg/kg) every 48 h, respectively, all via i.p injections. Mice were monitored daily over a 6 weeks period from the initial immunization for signs of arthritis. Standard scoring system for the severity of arthritis and statistical analysis of arthritis scores followed the previous method^18^. The severity of arthritis in each mouse was determined blindly and independently by four observers, and average of scores was calculated.

Infrared thermography and images of radiographs of the hind paws of the CIA mice on day 41 were captured with a FLIR T600 Thermal Imaging Camera and a Kodak Point-of-Care CR140 medical X-ray system, respectively. Analysis of regional temperature was carried out by XJ-Infrared report analysis software. The severity of bone destruction in the ankle joint was obtained according to the standard scoring system^33^, which was followed with 0, no swelling or bone damage; 1, slight ankle swelling or joint space narrowing; 2, moderate bone erosion and ankle swelling; and 3, severe bone erosion. The severity of arthritis in each mouse was determined blindly and independently by four observers, and average of scores was calculated.

## Electronic supplementary material

Supplementary Information
