# Supplementary material for: Trivalent soluble TNF Receptor, a potent TNF-α antagonist for the treatment collagen-induced arthritis
Source: Sci Rep. 2018 May 9;8:7327. doi: 10.1038/s41598-018-25652-w (PMC5943350; doi:10.1038/s41598-018-25652-w)
Supplement: Supplementary file 1 — Supplementary Information [file 41598_2018_25652_MOESM1_ESM.doc]

**Supplementary Information**

**Trivalent** **soluble TNF Receptor, a potent TNF-α antagonist for the treatment collagen-induced arthritis**

Xiaofang Cui, Linmo Chang, Youwei Li, Qianrui Lv, Fei Wang, Yaxian Lin, Weiyang Li, Jonathan D. Meade, Jamie C. Walden & Peng Liang

**Method**

**Histological analysis *and in situ* tissue staining for ligand-receptor affinity**

Hind paw samples from the animals in the CIA studies on day 41 were obtained and fixed in 4% paraformaldehyde, paraffin-embedded, and sectioned at 5μm thickness. For *in situ* ligand-receptor affinity tissue staining, the sections were rehydrated with ethanol of gradient concentration and stained with 1 U/ml of corresponding AP-TNF-α or AP alone as previously described. For hematoxylin and eosin (H&E) staining, the slides were sequentially stained with hematoxylin and eosin following the manufacturer’s instrunctions (Beyotime Biotechnology, Shanghai, China).

The internal organ tissues for toxicity evaluation were obtained from mice in TNFRII-Trimer treated group and normal control group. After being fixed with 10% neutral-buffered formalin and paraffin-embedded, sections (5μm) were cut from internal organ tissues. The internal organ sections were stained with hematoxylin and eosin (H&E) for the morphological examination of tissue cells. All tissue-staining images were captured with an Olympus BX53 upright microscope.

**Human hepatotoxicity assay**

The normal human liver cell line, LO_2_, was purchased from Shanghai Institute of Cell Biology (Shanghai, China) and cultured in RPMI-1640 with 10% FBS and 2mmol/L glutamine. LO_2_ cells were incubated with various concentrations of TNFRII-Trimer at 37°C for 24 hours, and cell viability was determined using tetrazolium (MTT) colorimetric test. Representative images were taken by phase contrast microscopy, and presented data were from representative experiments of at least 3 independent assays. Cleavage of PARP in LO_2_ cells was measured by western blot.


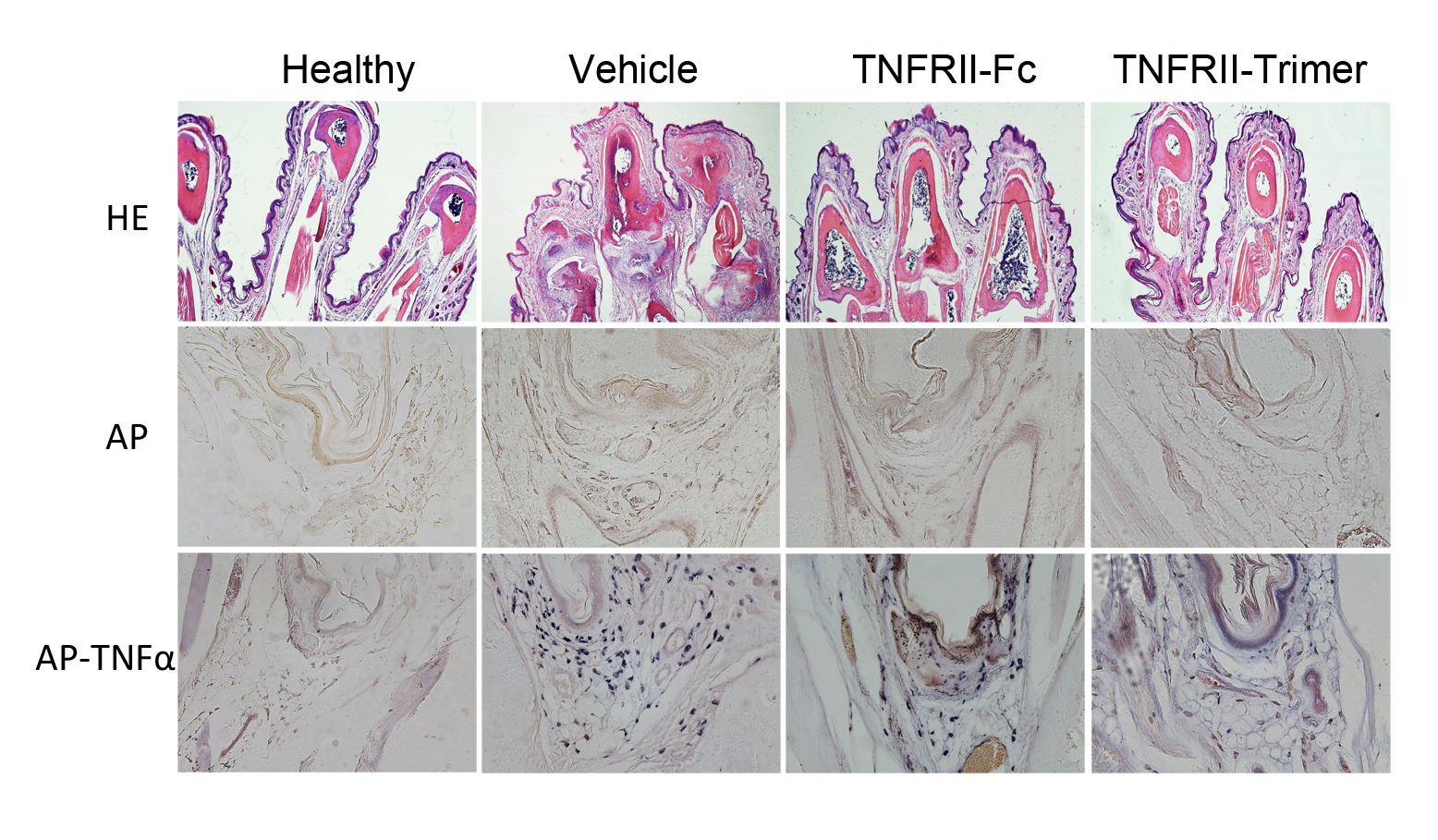


**Supplementary Fig. 1** TNFRII-Trimer significantly reduces the number of TNFR-positive immune infiltrates in DBA/1 mouse CIA model. Representative paraffin-embedded tissue sections from hind paws of mice mock treated (vehicle)，both anti-TNFα treatment groups as marked and normal control (healthy) were analyzed by both H&E staining (original magnification 4X) and *in situ* receptor affinity staining with AP-TNF-α and AP alone as negative control (original magnification 20X), respectively to detect TNFR-positive infiltrating macrophages. Large numbers of immune infiltrates over expressing TNFR were seen (blue color cells) in the disease tissues from the swollen paws vehicle treated mice, but not in normal controls. Both TNFRII-Fc and TNFRII-Trimer significantly reduced the number of TNFR-positive immune infiltrates. The results were representative of multiple independent experiments.


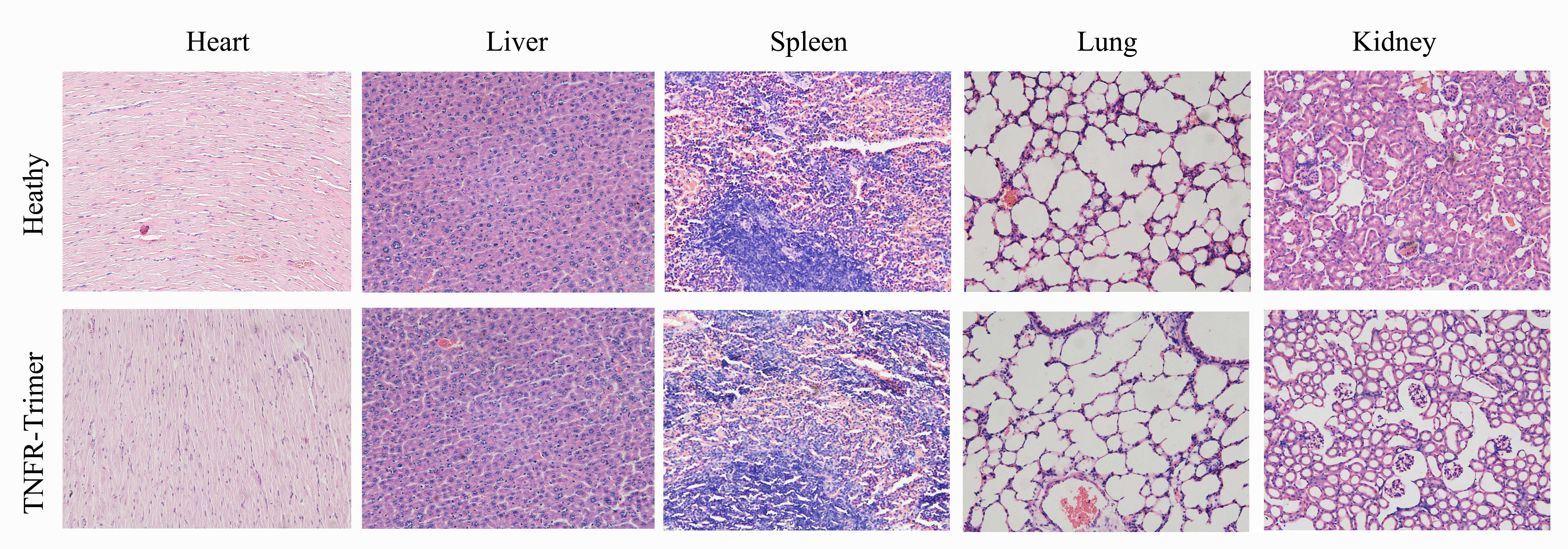


**Supplementary Fig. 2** TNFRII-Trimer lacks any toxicity. Representative H&E staining (original magnification 20X) of internal organs tissues from CIA mice treated with TNFRII-Trimer or vehicle mock treated healthy controls on day 41 at the end of the CIA experiment were shown. Compared with the healthy control, [no](../../AppData/Local/youdao/dict/Application/7.2.0.0703/resultui/dict/) apparent morphological [abnormalit](../../AppData/Local/youdao/dict/Application/7.2.0.0703/resultui/dict/)ies were seen in DBA/1 mice treated with TNFRII-Trimer.


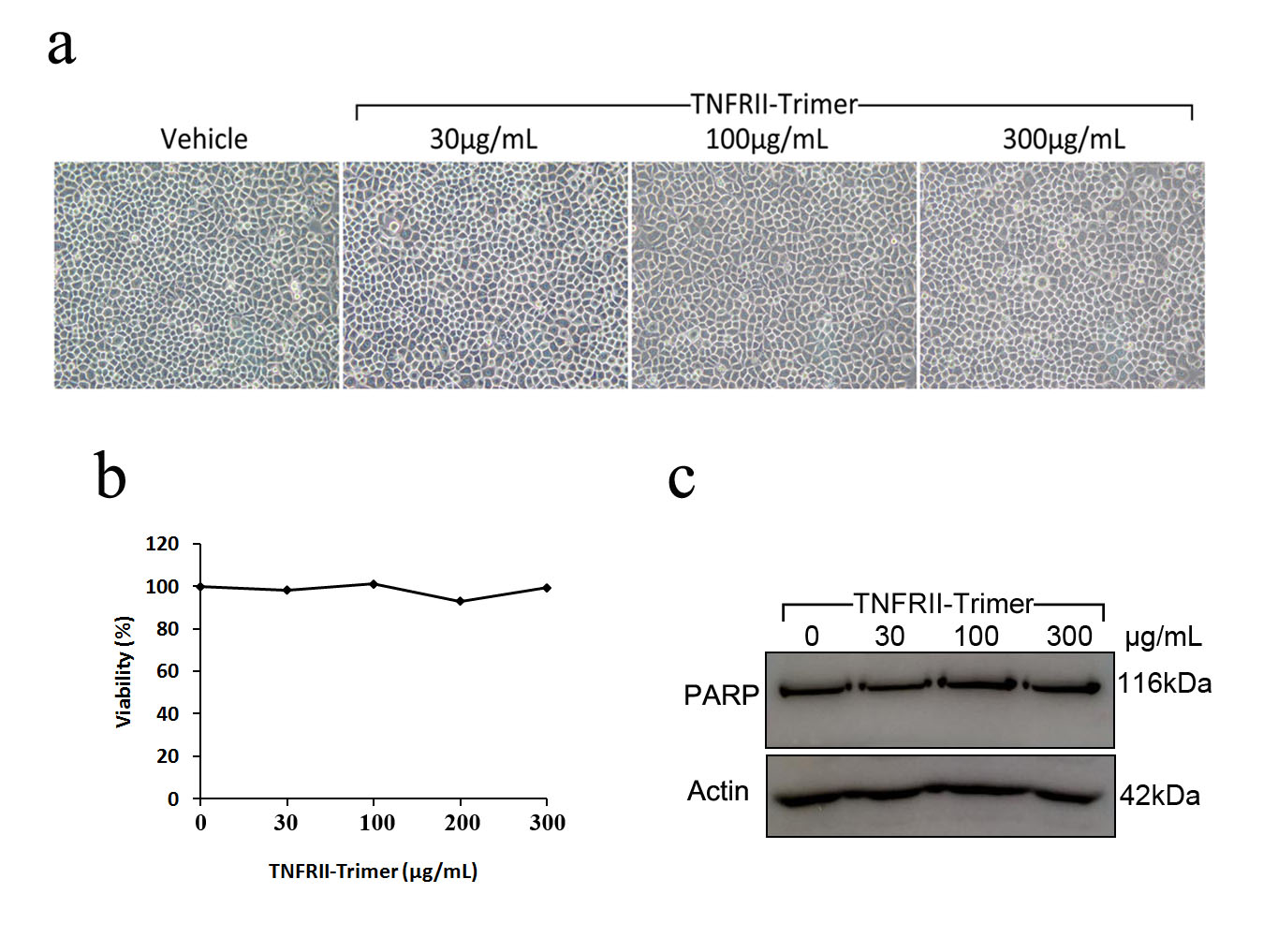


**Supplementary Fig. 3** TNFRII-Trimer lacks any hepatotoxicity for normal human hepatocytes. (a) Phase contrast images (×20) of human hepatocytes (LO_2_) taken after 24 h treatment with increasing [concentrations](../../AppData/Local/youdao/dict/Application/7.2.0.0703/resultui/dict/) of TNFRII-Trimer. (b) Cell viability of hepatocytes (LO_2_) treated for 24 h with TNFRII-Trimer were analyzed by MTT cell survival assay. (c) Apoptosis marker PARP cleavage were analyzed by Western blot after hepatocytes (LO_2_) were treated with [various](../../AppData/Local/youdao/dict/Application/7.2.0.0703/resultui/dict/) [concentrations](../../AppData/Local/youdao/dict/Application/7.2.0.0703/resultui/dict/) of TNFRII-Trimer *in vitro* after 24 h, and actin was used as a control for equal loading.

1 Xu, Y. *et al.* Functional Detection of TNF Receptor Family Members by Affinity-Labeled Ligands. *Sci Rep* **7**, 6944, doi:10.1038/s41598-017-06343-4 (2017).

2 Liu, X. *et al.* A Broad Blockade of Signaling from the IL-20 Family of Cytokines Potently Attenuates Collagen-Induced Arthritis. *Journal of immunology* **197**, 3029-3037, doi:10.4049/jimmunol.1600399 (2016).
